# Integrating Pharmacomechanical Treatments for Pulmonary Embolism Management within a Hub-and-Spoke System in the Swiss Ticino Region

**DOI:** 10.3390/jcm13092457

**Published:** 2024-04-23

**Authors:** Gianluca Guarnieri, Filip David Constantin, Giovanni Pedrazzini, Maria Antonella Ruffino, Daniel Sürder, Roberta Petrino, Enrico Carlo Zucconi, Luca Gabutti, Adam Ogna, Brenno Balestra, Marco Valgimigli

**Affiliations:** 1 Cardiocentro Ticino Institute, Ente Ospedaliero Cantonale, 6900 Lugano, Switzerland; gianluca.guarnieri@unimi.it (G.G.); filipdavid.constantin@eoc.ch (F.D.C.); giovanni.pedrazzini@eoc.ch (G.P.); daniel.surder@eoc.ch (D.S.); 2 Department of Clinical Sciences and Community Health, University of Milan, 20122 Milan, Italy; 3 Ospedale Regionale di Lugano, Ente Ospedaliero Cantonale, 6900 Lugano, Switzerland; mariaantonella.ruffino@eoc.ch (M.A.R.); roberta.petrino@eoc.ch (R.P.); enricocarlo.zucconi@eoc.ch (E.C.Z.); 4 Ospedale Regionale di Bellinzona e Valli, Ente Ospedaliero Cantonale, 6500 Bellinzona, Switzerland; luca.gabutti@eoc.ch; 5 Ospedale Regionale di Locarno, Ente Ospedaliero Cantonale, 6600 Locarno, Switzerland; adam.ogna@eoc.ch; 6 Ospedale Regionale di Mendrisio, Ente Ospedaliero Cantonale, 6850 Mendrisio, Switzerland; brenno.balestra@eoc.ch

**Keywords:** pulmonary embolism, thrombolysis, FlowTriever, catheter-directed thrombolysis, PERT, EKOS

## Abstract

The Swiss Ticino regional pulmonary embolism response team (PERT) features direct access to various pharmacomechanical PE management options within a hub/spoke system, by integrating evidence, guidelines’ recommendations and personal experiences. This system involves a collaborative management of patients among the hospitals distributed throughout the region, which refer selected intermediate–high or high PE patients to a second-level hub center, located in Lugano at Cardiocentro Ticino, belonging to the Ente Ospedaliero Cantonale (EOC). The hub provides 24/7 catheterization laboratory activation for catheter-based intervention (CBI), surgical embolectomy and/or a mechanical support system such as extracorporeal membrane oxygenation (ECMO). The hub hosts PE patients after percutaneous or surgical intervention in two intensive care units, one specialized in cardiovascular anesthesiology, to be preferred for patients without relevant comorbidities or with hemodynamic instability and one specialized in post-surgical care, to be preferred for PE patients after trauma or surgery or with relevant comorbidities, such as cancer. From April 2022 to December 2023, a total of 65 patients were referred to the hub for CBI, including ultrasound-assisted catheter-directed thrombolysis (USAT) or large-bore aspiration intervention. No patient received ECMO or underwent surgical embolectomy.

## 1. Background and 2019 European Society of Cardiology Guidelines

Venous thromboembolism (VTE), encompassing deep vein thrombosis (DVT) and pulmonary embolism (PE), is the third leading cause of cardiovascular disease after myocardial infarction and stroke. Despite improvements in the pharmacological management of this condition, PE is still associated with significant mortality and morbidity [1]. The case fatality rate varies widely, but approximately 10% of all patients with acute pulmonary embolism die within 3 months after the diagnosis [2].

Time trend analyses in European, Asian and North American populations suggest that case fatality rates of acute PE are decreasing [3,4,5,6]. This might be a reflection of more effective and timely initiation of therapy. However, there is also a tendency towards the overdiagnosis of (subsegmental or even non-existent) PE in the modern era [7], and this might in turn lead to a false drop in case fatality rates by inflating the denominator, i.e., the total number of PE cases.

Acute PE interferes with both circulation and gas exchange. Right ventricular (RV) failure due to acute pressure overload is considered the primary cause of death in PE.

The risk stratification of patients with acute PE is mandatory for determining the appropriate therapeutic management approach [8].

The presence of clinical parameters of PE severity, such as RV dysfunction and/or elevated cardiac levels (intermediate-risk PE) or hemodynamic instability (high-risk PE), is associated with a worse prognosis [8].

Patients with confirmed PE and hemodynamic instability fulfill the high-risk criteria. In these patients, the 2019 ESC guidelines provide three Class I recommendations, consisting of anticoagulation with unfractionated heparin (UFH), including a weight-adjusted bolus injection (Level of evidence [LOE] C), systemic thrombolytic therapy, if not contraindicated (LOE B), and surgical pulmonary embolectomy, if thrombolysis is contraindicated or has failed (LOE C) [8].

The single evidence-based Class I recommendation is supported by a study-level meta-analysis of fifteen trials involving 2057 patients. Compared with heparin, thrombolytic therapy was associated with a significant reduction in overall mortality (OR; 0.59, 95% CI: 0.36–0.96). This reduction was not statistically significant after the exclusion of studies including high-risk PE (OR; 0.64, 95% CI: 0.35–1.17). Major hemorrhage (OR; 2.91, 95% CI: 1.95–4.36) and fatal or intracranial bleeding (OR: 3.18, 95% CI: 1.25–8.11) were significantly more frequent among patients receiving thrombolysis [9]. Only four studies assessed the benefits and risks of systemic thrombolytic therapy in high-risk PE, of which three included a heterogenous population of intermediate- and high-risk PE, and a single trial of eight patients included a pure high-risk PE population [9]. The single most important trial on systemic thrombolytic therapy for PE management is the double-blind PEITHO trial, which randomized 1006 normotensive (i.e., high-risk PE was an exclusion criterion) patients with intermediate-risk PE to tenecteplase plus heparin versus placebo plus heparin. The use of thrombolytic therapy was associated with lower all-cause mortality and hemodynamic decompensation compared with the use of anticoagulant alone (2.6% vs. 5.6%, Odds Ratio [OR] 0.44; 95% confidence interval [CI] 0.23–0.87). Death from any cause at day 7 did not differ (1.2% vs. 1.8%, OR 0.65; 0.23–1.85), whereas the benefit for the primary composite endpoint was almost exclusively driven by hemodynamic decompensation, which was defined as the need for cardiopulmonary resuscitation; or systolic blood pressure < 90 mm Hg for at least 15 min, or a drop in systolic blood pressure by at least 40 mm Hg for at least 15 min with signs of end-organ hypoperfusion (cold extremities or low urinary output < 30 mL/h or mental confusion); or the need for catecholamine administration to maintain adequate organ perfusion and a systolic blood pressure of >90 mm Hg (including dopamine at a rate of >5 micrograms/kg per minute) [10].

Tenecteplase was associated with a fivefold increase in major extracranial bleeding (6.3% vs. 1.2%, OR 5.55, 95% CI 2.3–13.39, *p* < 0.001) and a more than tenfold increase in stroke (2.4% vs. 0.2%, OR 12.10, 95% CI 1.57–93.39, *p* = 0.003), mainly due to hemorrhagic events [10]. The high rate of bleeding, including intracranial hemorrhages, has dampened clinician enthusiasm for full-dose systemic fibrinolysis in patients without hemodynamic instability and, despite the paucity of data, the latter regimen has been reserved to high-risk PE by current guidelines.

In the last decade, catheter-based interventions are being developed for intermediate- or high-risk PE [11], with more acceptable bleeding risk, especially intracranial bleeding [12]. Most knowledge about catheter-based embolectomy is derived from registries and pooled results from case series.

Catheter-directed thrombolysis (CDT) is usually administered through a catheter with multiple sideholes and direct thrombolytic infusion into the pulmonary arteries. Additionally, ultrasound-assisted catheter-directed thrombolysis (USAT) represents a novel pharmacomechanical thrombolysis treatment that combines CDT and ultrasound energy to improve the delivery of thrombolytic agents [13]. More recently, large-bore aspiration catheters, such as the 24-French FlowTriever (INARI), have gained popularity across trials and practice, especially in the United States, allowing for direct thrombus suction without the need for regional thrombolysis.

According to the 2019 ESC guidelines, percutaneous catheter-directed treatment should be considered for patients with high-risk PE, in whom thrombolysis is contraindicated or has failed or for patients with hemodynamic deterioration on anticoagulation treatment (Class IIa, OE C) [8].

Finally, the set-up of PE response teams (PERTs) is encouraged by recent guidelines, as they address the needs of modern system-based healthcare. A PERT brings together a team of specialists from different disciplines including, for example, cardiology, pulmonology, hematology, emergency medicine, vascular medicine, anesthesiology/intensive care, cardiothoracic surgery and (interventional) radiology. The team convenes in real time (face-to-face or via web conference) to enhance clinical decision making. The exact composition and operating mode of a PERT are not fixed, depending on the resources and expertise available in each hospital for the management of acute PE.

## 2. Canton Ticino’s Protocol for Pulmonary Embolism Management

The prompt access to different therapeutic options requires the presence of a regional network among hospitals, allowing for access to more specialized treatments based on patient risk profiles.

A hub/spoke system has been developed for the management of PE in Canton Ticino. This system involves a collaborative management of patients among the hospitals distributed throughout the region, which refer selected PE patients to a single second-level hub center, located in Lugano. The hub center includes the Cardiocentro Ticino Institute with the support of the facilities at Civico Hospital, as part of the EOC. The hub provides 24/7 catheterization laboratory activation for catheter-based intervention (CBI) and/or a mechanical support system such as extracorporeal membrane oxygenation (ECMO) or surgical embolectomy. The hub hosts PE patients after percutaneous or surgical intervention in two intensive care units, one located at Cardiocentro Ticino and specialized in cardiovascular anesthesiology is preferred for PE patients without relevant comorbidities or with hemodynamic instability, and one located at Civico Hospital, specialized in post-surgical care, is preferred for PE patients after trauma or surgery or with relevant comorbidities, such as cancer.

**Figure 1:**
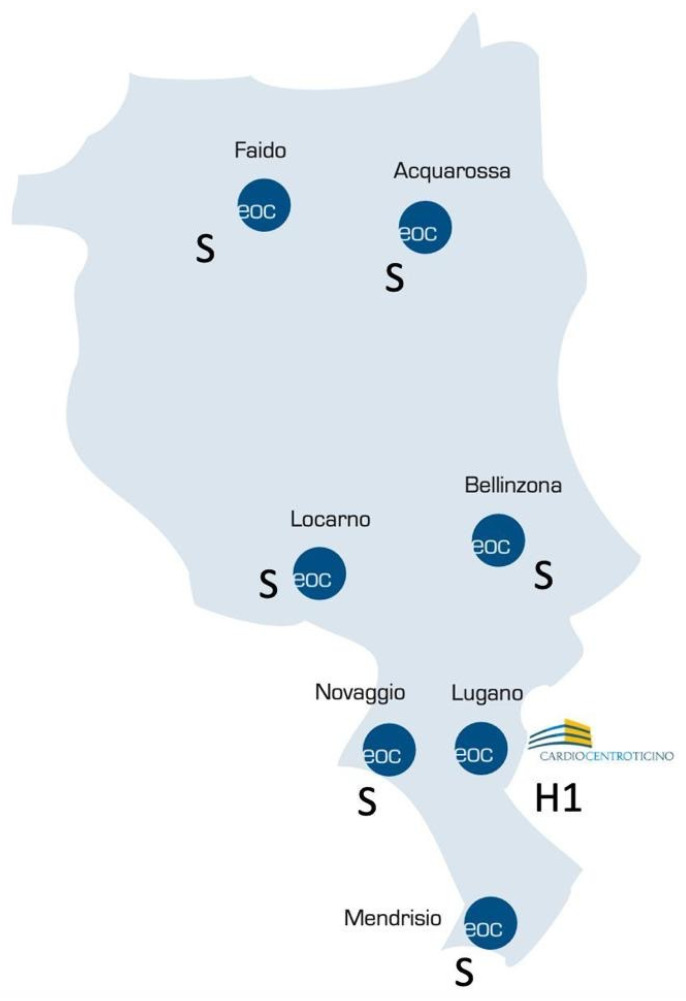
The hub/spoke system for PE in Canton Ticino. Cardiocentro Ticino is the hub (H1) center, while the other hospitals are spokes (S).

A map illustrating the organization of the hub-and-spoke system of the Canton Hospital Authority is depicted in Figure 1.

In keeping with the ESC guidelines, our protocol has identified and integrated various PERT members for the decision making of the most suitable treatment option for each notified PE case. The PERT is activated for intermediate–high- or selected high-risk PE by the notifying physician (i.e., an emergency physician or other physicians overseeing the patient if pulmonary embolism occurs or is detected after hospital admission) with a phone call to the on-duty cardiologist, who then discusses the case with the interventional cardiologist, the intensivist and the cardio-anesthesiologist. If CBI is planned, the invasive team is composed of an interventional cardiologist (constitutive operator) and an interventional radiologist (optional operator). The cardiothoracic surgeon is an optional PERT member to be consulted in selected high-risk PE for surgical embolectomy.

**Figure 2:**
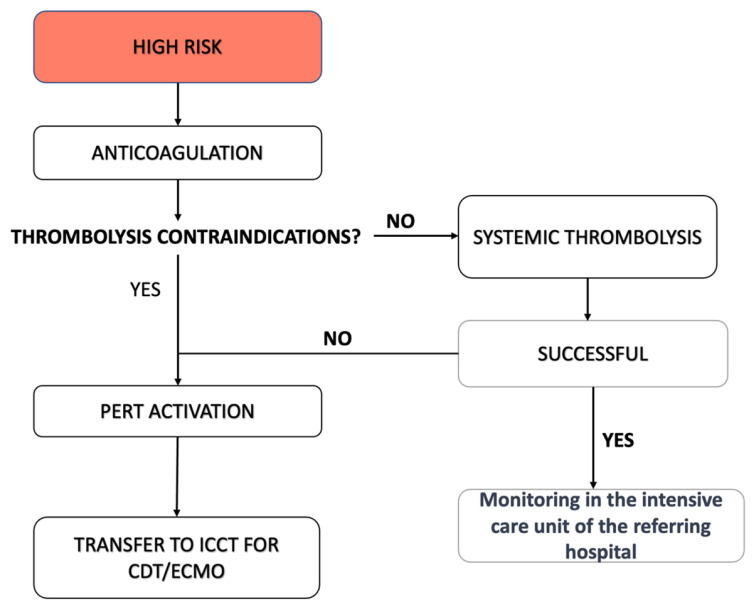
Management of high-risk PE, according to our protocol.

In the case of high-risk PE, our regional protocol encourages immediate systemic thrombolysis, if not contraindicated, without the need to activate the PERT or centralize the patient (Figure 2). Thrombolytic therapy (10 mg bolus of alteplase administered in 1–2 min, followed by 90 mg infusion over 120 min) is administered concurrently with a continuous infusion of sodium heparin. One hour after the start of treatment, the PERT is consulted if hemodynamic conditions do not improve. Similarly, the PERT is consulted directly in high-risk PE if thrombolysis is contraindicated in view of CBI and/or ECMO.

**Figure 3:**
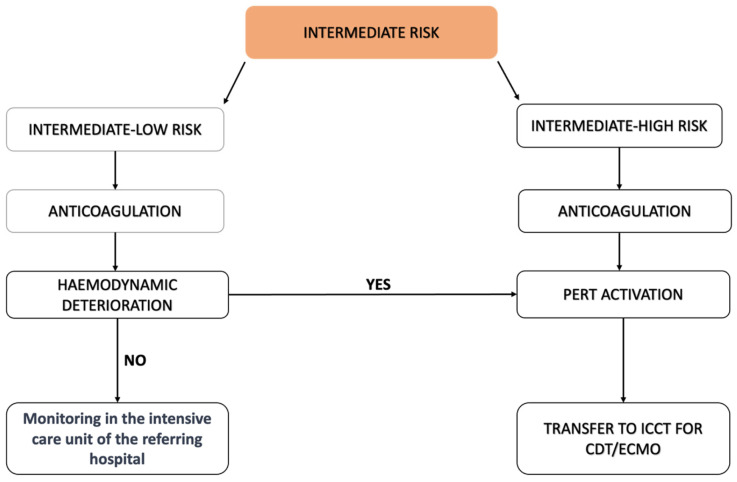
Management of intermediate-risk PE, according to our protocol.

Our protocol encourages PERT activation for intermediate–high-risk PE. Intermediate–low-risk PE (i.e., troponin-positive patients without signs of RV dysfunction at CT scan and/or at transthoracic echocardiogram) is managed with anticoagulant therapy only. Intermediate–high-risk PE is similarly managed with UFH administration but is considered for immediate transfer to the hub for CBI (Figure 3). Therefore, our protocol offers CBI as a first-line add-on treatment to standard anticoagulation, not as a second-line treatment.

Among CBI, USAT with EKOS^®^ has been the first treatment option adopted within our protocol, since its inception, in April 2022, whereas since July 2023, the FlowTriever INARI system has been implemented in selected cases by two expert operators. From April 2022 to December 2023, a total of 65 patients have been treated with CBI.

We adopt as standard protocol with USAT/EKOS the 6 h regimen, according to which 1 mg/h alteplase is administered through each catheter. This regimen has been selected based on the OPTALYSE PE trial, which showed a significant reduction in pulmonary thrombosis at CT scan and no episodes of intracranial bleeding.

Following this 6 h interval, an echocardiogram is performed to re-assess RV function. Should no improvements be observed, the catheters are retained in situ for an additional 9 h, and additional local lytic treatment is administered at a reduced rate of 0.5 mg/h, in keeping with the ULTIMA protocol [14].

The FlowTriever system has been implemented more recently as CBI under the rationale that there are very high bleeding risk patients with intermediate–high PE who are not eligible to low-dose lysis. In addition, the FlowTriever system acts by direct thrombus aspiration and is being assessed as a suitable CBI for high-risk PE, should these patients be ineligible for lysis.

After CBI, an echocardiogram is repeated to re-assess RV function and diameters. Similarly, as the internal quality standard, we perform a follow-up CT scan at 48 h in all patients without chronic kidney dysfunction.

To further illustrate our protocol, we report the single patient in our case series who underwent two sequential CBIs with USAT/EKOS followed by the FlowTriever system.

A 75-year-old patient, without significant medical history, self-presented to the emergency department of Civico regional Hospital with acute dyspnea 24 h following a 6 h road trip. The onset of dyspnea occurred suddenly with minimal exertion, accompanied by dizziness and malaise. The hemodynamic status was stable with blood systolic pressure around 135 mmHg and sinus tachycardia (HR 120 bpm). The respiratory rate was 22 bpm, and peripheral saturation was 93%.

An arterial blood sample showed mild hypoxemia and mild hypocapnia (pCO2 3.9 kPa, reference values 4.67–6.4 kPa and pO2 7.8 kPa, reference values 11.1–14.4), with a pH within the normal range (pH 7.43, reference values 7.35–7.45), reduced peripheral oxygen saturation (SatO2 90%) and a slightly increased serum lactate (Lac 2.6 mmol/L, reference values < 2 mmol/L).

Blood testing showed troponin elevation (high-sensitivity troponin T, hsTn was 39 ng/L, reference values < 14 ng/L), whereas NT-pro BNP was 3500 ng/L (reference values < 879 ng/L).

The ECG showed sinus tachycardia but otherwise was unremarkable.

A color Doppler ultrasound of the lower limbs showed the presence of bilateral proximal deep vein thrombosis involving the common femoral vein on the right and the superficial femoral vein with the popliteal vein on the left.

The thorax CT angiography performed revealed a bilateral central pulmonary embolism involving the right and left branches of the pulmonary artery with acute pulmonary heart disease. The patient was subsequently transferred to our center for USAT using the EKOS system. The treatment protocol involved an initial 6 h infusion of alteplase. At 6 h, transthoracic echocardiography showed the persistence of RV dilatation, therefore alteplase infusion was continued for an additional 9 h at halved-regimen.

**Figure 4:**
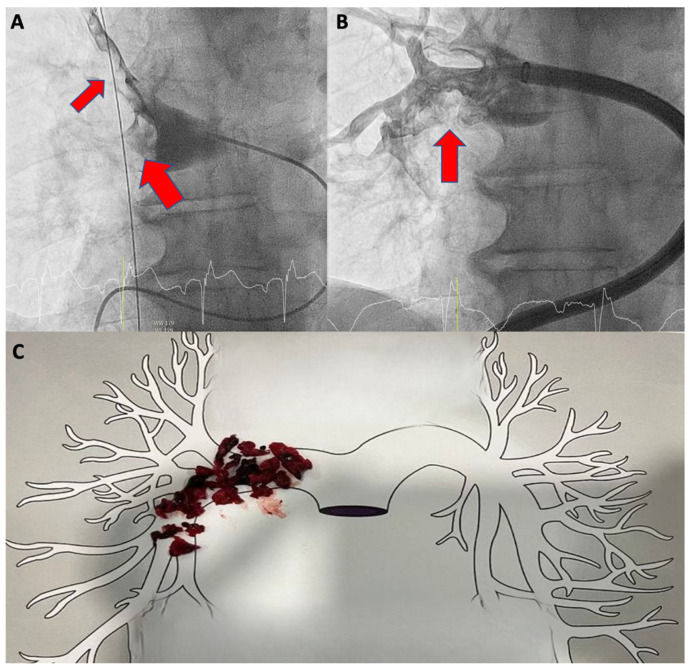
Selective pulmonary angiography of the right pulmonary artery conducted during before (**A**) and after the FlowTriever (**B**). The red narrows show the presence of thrombotic clots in right pulmonary artery. In (**C**), the aspirated thrombotic removal with FlowTriever is illustrated in a schematic diagram of the major vessels in the pulmonary circuit.

On day 2, a second transthoracic echocardiogram revealed persistent RV dilatation (areaDi = 15.5 cmq/sq m, basal diameter = 50 mm) with reduced radial function (FAC 31%). The follow-up thorax CT angiogram showed persistent and largely unmodified bilateral central pulmonary thrombosis with complete right pulmonary artery obstruction. We opted to use the FlowTriever System INARI as rescue therapy. The corresponding aspirated material in the right pulmonary artery main trunk is shown in Figure 4.

**Figure 5:**
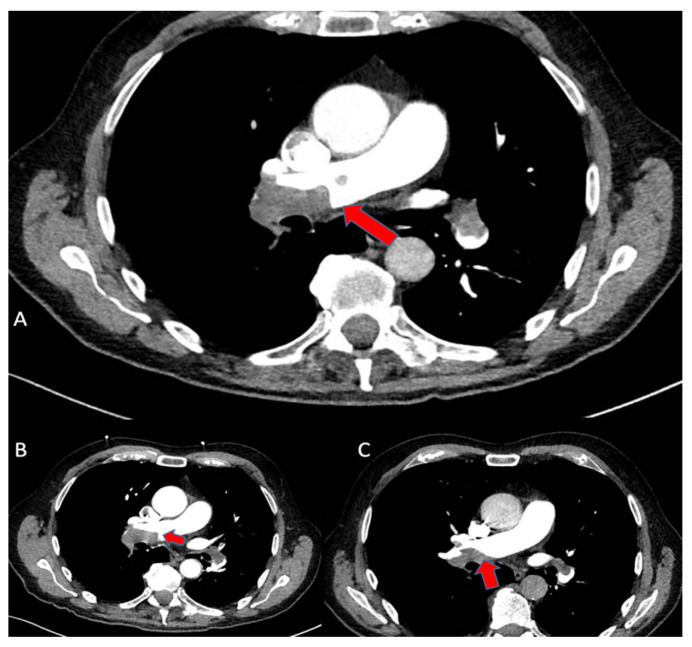
(**A**): CT scan at baseline showing PE; (**B**): CT scan performed after EKOS treatment showing very limited reduction in emboli; (**C**): CT scan showing a significant reduction in thrombotic burden after thromboaspiration with FlowTriever. The red arrows show the thrhombotic formations in the right pulmonary artery.

On day 3 (24 h after the use of FlowTriever), a third thorax CT angiogram showed mild thrombus dissolution at the segmental branches of the right lower pulmonary lobe. A sequence of CT scans is shown in Figure 5.

The transthoracic echocardiogram after the use of FlowTriever revealed some improvement in RV dilatation (areaDi = 14 cmq/mq, basal diameter = 48 mm) and radial function (FAC = 33%). The patient was then discharged without complications under direct oral anticoagulation.

CBI is being developed to offer a safer alternative to systemic lytic treatment to reduce RV overload by reducing thrombus load in pulmonary circulation. While anticoagulation is key to stop thrombus formation and subsequent embolization, thrombus lysis relies entirely on endogenous protease tissue-type plasminogen activator (tPA), which requires time and is frequently unable to entirely dissolve large emboli. In the hope to improve the short- and long-term outcomes of intermediate–high- and even more high-risk PE, CBI may prove to be more effective than oral anticoagulation alone or oral anticoagulation and systemic lytic treatment, respectively. Epidemiological studies indicate that in more than 50% of cases, pulmonary obstruction may persist 3 months after standard oral anticoagulation, which might result in chronic pulmonary thromboembolism (CPTE) disease and in chronic thromboembolic pulmonary hypertension (CTEPH). Sanchez et al. demonstrated that up to 29% of patients had defects of perfusion at 6 to 12 months after PE [15]. Several risk factors have been identified in the development of CTEPH, pre-existing conditions such as pulmonary hypertension or RV compromise, a high thrombus load, diagnostic delay, failure to achieve complete thrombus resolution, recurrent symptomatic PE and the location of emboli in the central pulmonary artery. The early diagnosis of this condition remains challenging, often taking an average of 14 months from the onset of symptoms to diagnosis in specialized medical centers. Liu et al. found that the protein S activity, S_d_/MPA_d_ (the ratio of the sum of the residual segmental pulmonary artery diameter to the main pulmonary artery diameter at CT scan), and the location of emboli at the moment of PE diagnosis were independent risk factors for the chronic persistence of thromboembolism following the acute event [16]. Moreover, the treatment with fibrinolytic agents at the time of the initial presentation has not definitively shown improvements in the frequency or extent of the recovery of pulmonary vascular perfusion [17]. Therefore, interventional percutaneous therapies may also play a role in the prevention of this condition in the case of high-risk patients.

Randomized control studies are running and will ultimately inform the community on the role of CBI for the management of higher-risk PE.

## Data Availability

The data presented in this study are available on request from the corresponding author.

## References

[1] Turetz M., Sideris A.T., Friedman O.A., Triphathi N., Horowitz J.M. (2018). Epidemiology, Pathophysiology, and Natural History of Pulmonary Embolism. Semin. Interv. Radiol..

[2] Aujesky D., Jiménez D., Mor M.K., Geng M., Fine M.J., Ibrahim S.A. (2009). Weekend versus weekday admission and mortality after acute pulmonary embolism. Circulation.

[3] Keller K., Hobohm L., Ebner M., Kresoja K.-P., Münzel T., Konstantinides S.V., Lankeit M. (2020). Trends in thrombolytic treatment and outcomes of acute pulmonary embolism in Germany. Eur. Heart J..

[4] Lehnert P., Lange T., Møller C.H., Olsen P.S., Carlsen J. (2018). Acute Pulmonary Embolism in a National Danish Cohort: Increasing Incidence and Decreasing Mortality. Thromb. Haemost..

[5] Jiménez D., de Miguel-Díez J., Guijarro R., Trujillo-Santos J., Otero R., Barba R., Muriel A., Meyer G., Yusen R.D., Monreal M. (2016). Trends in the Management and Outcomes of Acute Pulmonary Embolism: Analysis from the RIETE Registry. J. Am. Coll. Cardiol..

[6] Agarwal S., Clark D., Sud K., Jaber W.A., Cho L., Menon V. (2015). Gender Disparities in Outcomes and Resource Utilization for Acute Pulmonary Embolism Hospitalizations in the United States. Am. J. Cardiol..

[7] Wiener R.S., Schwartz L.M., Woloshin S. (2011). Time trends in pulmonary embolism in the United States: Evidence of overdiagnosis. Arch. Intern. Med..

[8] Konstantinides S.V., Meyer G., Becattini C., Bueno H., Geersing G.J., Harjola V.P., Huisman M.V., Humbert M., Jennings C.S., Jiménez D. (2020). 2019 ESC Guidelines for the diagnosis and management of acute pulmonary embolism developed in collaboration with the European respiratory society (ERS). Eur. Heart J..

[9] Marti C., John G., Konstantinides S., Combescure C., Sanchez O., Lankeit M., Meyer G., Perrier A. (2015). Systemic thrombolytic therapy for acute pulmonary embolism: A systematic review and meta-analysis. Eur. Heart J..

[10] Meyer G., Vicaut E., Danays T., Agnelli G., Becattini C., Beyer-Westendorf J., Bluhmki E., Bouvaist H., Brenner B., Couturaud F. (2014). Fibrinolysis for patients with intermediate-risk pulmonary embolism. N. Engl. J. Med..

[11] Nagraj S., Li W., Zamora C., Barakakis P.A., Kokkinidis D.G. (2022). Pharmacological and interventional management of pulmonary embolism: Where do we stand?. Future Cardiol..

[12] Rousseau H., Del Giudice C., Sanchez O., Ferrari E., Sapoval M., Marek P., Delmas C., Zadro C., Revel-Mouroz P. (2021). Endovascular therapies for pulmonary embolism. Heliyon.

[13] Götzinger F., Lauder L., Sharp A.S.P., Lang I.M., Rosenkranz S., Konstantinides S., Edelman E.R., Böhm M., Jaber W., Mahfoud F. (2023). Interventional therapies for pulmonary embolism. Nat. Rev. Cardiol..

[14] Kucher N., Boekstegers P., Müller O.J., Kupatt C., Beyer-Westendorf J., Heitzer T., Tebbe U., Horstkotte J., Müller R., Blessing E. (2014). Randomized, controlled trial of ultrasound-assisted catheter-directed thrombolysis for acute intermediate-risk pulmonary embolism. Circulation.

[15] Sanchez O., Helley D., Couchon S., Roux A., Delaval A., Trinquart L., Collignon M., Fischer A., Meyer G. (2010). Perfusion defects after pulmonary embolism: Risk factors and clinical significance. J. Thromb. Haemost..

[16] Liu W., Xie S., Liang T., Chang F., Liu M., Zhai Z. (2022). Clinical and imaging risk factors for the persistence of thromboembolism following acute pulmonary embolism. Quant. Imaging Med. Surg..

[17] Fernandes T., Planquette B., Sanchez O., Morris T. (2016). From Acute to Chronic Thromboembolic Disease. Ann. Am. Thorac. Soc..

